# Adamantane-1-thio­amide

**DOI:** 10.1107/S1600536809027470

**Published:** 2009-07-18

**Authors:** Maryam Zahid, M. Khawar Rauf, Michael Bolte, Shahid Hameed

**Affiliations:** a Department of Chemistry, Quaid-i-Azam University, Islamabad 45320, Pakistan; b Institut für Anorganische Chemie, J. W. Goethe-Universität Frankfurt, Max-von-Laue-Strasse 7, 60438 Frankfurt/Main, Germany

## Abstract

The title compound, C_11_H_17_NS, is an important inter­mediate for the synthesis of biologically active adamantlythia­zolo-oxadiazo­les. The adamantyl residue is disordered about a twofold rotation axis over two sites with site-occupation factors of 0.817 (3) and 0.183 (3). The crystal structure is stabilized by inter­molecular N—H⋯S hydrogen-bonding inter­actions.

## Related literature

Adamantane derivatives include well known drugs such as Rimantadine, Memantine, Adapalene and Adatanserin, see: Krasnikov *et al.* (2004 ▶). For their biological activity, see: Singh *et al.* (2007 ▶); Wennekes *et al.* (2007 ▶); Inaba *et al.* (2007 ▶); Kolocouris *et al.* (2007 ▶). Thio­amides are not only widely used as fungicides (Klimesova *et al.*, 1999 ▶) and herbicides (Bahadir *et al.*, 1979 ▶) but are also valuable inter­mediates in the synthesis of heterocyclic compounds (Jagodzinski, 2003 ▶). For the synthesis of the title compound, see: Kaboudin & Elhamifar (2006 ▶).
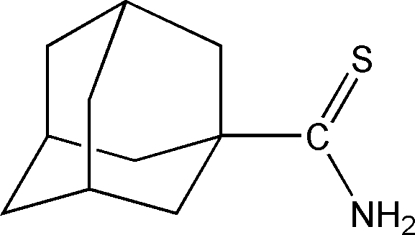


## Experimental

### 

#### Crystal data

C_11_H_17_NS
*M* *_r_* = 195.32
Monoclinic, 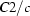

*a* = 24.255 (2) Å
*b* = 7.9879 (5) Å
*c* = 11.2928 (9) Å
β = 100.859 (7)°
*V* = 2148.8 (3) Å^3^
*Z* = 8
Mo *K*α radiation
μ = 0.26 mm^−1^
*T* = 173 K
0.39 × 0.26 × 0.25 mm

#### Data collection

Stoe IPDS-II two-circle diffractometer
Absorption correction: multi-scan (*MULABS*; Spek, 2009 ▶; Blessing, 1995 ▶) *T*_min_ = 0.907, *T*_max_ = 0.939
7423 measured reflections
2002 independent reflections
1703 reflections with *I* > 2σ(*I*)
*R*_int_ = 0.040

#### Refinement

*R*[*F* ^2^ > 2σ(*F* ^2^)] = 0.038
*wR*(*F* ^2^) = 0.103
*S* = 1.07
2002 reflections
163 parameters
35 restraints
H atoms treated by a mixture of independent and constrained refinement
Δρ_max_ = 0.20 e Å^−3^
Δρ_min_ = −0.30 e Å^−3^

### 

Data collection: *X-AREA* (Stoe & Cie, 2001 ▶); cell refinement: *X-AREA*; data reduction: *X-AREA*; program(s) used to solve structure: *SHELXS97* (Sheldrick, 2008 ▶); program(s) used to refine structure: *SHELXL97* (Sheldrick, 2008 ▶); molecular graphics: *XP* in *SHELXTL-Plus* (Sheldrick, 2008 ▶); software used to prepare material for publication: *SHELXL97*.

## Supplementary Material

Crystal structure: contains datablocks I, global. DOI: 10.1107/S1600536809027470/hg2536sup1.cif

Structure factors: contains datablocks I. DOI: 10.1107/S1600536809027470/hg2536Isup2.hkl

Additional supplementary materials:  crystallographic information; 3D view; checkCIF report

## Figures and Tables

**Table 1:** Hydrogen-bond geometry (Å, °)

*D*—H⋯*A*	*D*—H	H⋯*A*	*D*⋯*A*	*D*—H⋯*A*
N1—H1*A*⋯S1^i^	0.874 (9)	2.631 (13)	3.4027 (14)	147.9 (16)
N1—H1*B*⋯S1^ii^	0.870 (9)	2.492 (10)	3.3485 (14)	168.1 (17)

Symmetry codes: (i) 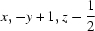; (ii) 
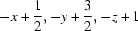
.

**Fig. 1.**
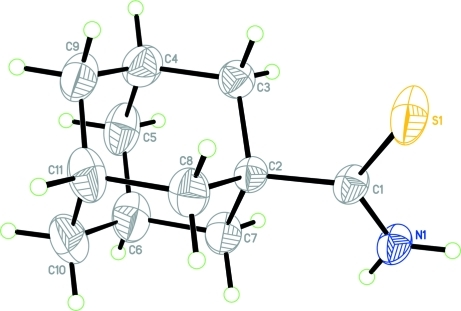
Molecular structure of the title compound (I) showing atom-labelling scheme and displacement ellipsoids at the 50% probability level. The disordered atoms of the minor occupied site have been omitted for clarity.

**Fig. 2.**
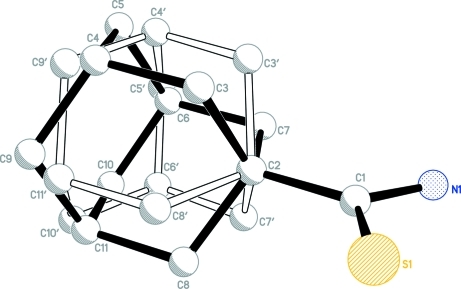
Molecular structure of the title compound (I) showing both the major and minor occupied positions of the disordered atoms.

## supplementary crystallographic information

### Comment

Adamantane derivatives have found widespread use as biologically active agents to combat various human pathogens. These derivatives include the well known drugs like Rimantadine, Memantine, Adapalene and Adatanserin (Krasnikov *et al.*, 2004). A broad spectrum of biological activities like antimalarial (Singh *et al.*, 2007), glucosylceramide metabolism inhibitors (Wennekes *et al.*, 2007), vitamin D receptor modulators (Inaba *et al.*, 2007) and anti-influenza (Kolocouris *et al.*, 2007), is associated with adamantane containing preparations and compounds. Thioamides, on the other hand, are not only widely used as fungicides (Klimesova *et al.*, 1999) and herbicides (Bahadir *et al.*, 1979) but are also valuable intermediates in the synthesis of heterocyclic compounds (Jagodzinski, 2003). The title compound, adamantane-1-thioamide (1), was synthesized in this laboratory as an intermediate in the synthesis of adamantlythiazolo-oxadiazoles to explore their potential as antitumour agents. The synthesis was accomplished by treating adamantane-1-carbonitrile with P_4_S_10_ according to a known procedure (Kaboudin *et al.*, 2006). Here, we are going to report the crystal structure of (1). The crystal structure is stabilized by intermolecular N—H···S, hydrogen-bond interactions.

### Experimental

A solution of P_4_S_10_ (3.1 g, 7.0 mmol.) in ethanol (10 ml) was stirred for 1 h. Adamantane-1-carbonitrile (0.5 g, 3.5 mmol.) was added and the mixture refluxed for 12 h. The mixture was concentrated, water (25 ml) was added and extracted with dichloromethane (3 × 25 ml). The combined organic extracts were dried (anhydrous Na_2_SO_4_, concentrated on rotary and refrigerated. The white precipitates separated were recrystallized from ethanol. Yield: 62%; m.p.: 159–162 °C; Rf: 0.40 (n-hexane: ethylacetate; 7:3); IR (ν_max_, KBr, cm^-1^): 3424, 3323, 3144, 2907, 2848, 1656, 1449, 1384, 1310, 1240; ^1^H-NMR (CDCl_3_): δ 7.9 (1*H*, b), 7.1 (1*H*, b), 1.9 (9*H*, b), 1.71 (6*H*, b); ^13^C-NMR (CDCl_3_): δ 218.8, 45.6, 41.7, 36.2, 28.4; EIMS: (*m*/*z* %) 195 (80), 162 (15), 135 (100), 107 (13),93 (20), 79 (23), 60 (13); Elemental analysis for C_11_H_17_NS (195.32):*C*, 67.64; H, 8.77; N, 7.17. Found: C, 67.87; H, 8.88; N, 7.38.

### Refinement

H atom on the N atom was refined isotropically. Other H atoms were placed in idealized positions and treated as riding atoms with C—H distances in the range 0.99–1.00 Å and *U*_iso_(H) = 1.2*U*_eq_(C). The adamantyl residue is disordered about a twofold rotation axis over two sites with site occupation factors of 0.817 (3) and 0.183 (3). Similarity restraints were applied to keep the bond lengths and angles of the minor occupied site in a reasonable range.

### Crystal data

**Table:**

C_11_H_17_NS	*F*(000) = 848
*M**_r_* = 195.32	*D*_x_ = 1.208 Mg m^−^^3^
Monoclinic, *C*2/*c*	Mo *K*α radiation, λ = 0.71073 Å
Hall symbol: -C 2yc	Cell parameters from 7109 reflections
*a* = 24.255 (2) Å	θ = 3.5–25.9°
*b* = 7.9879 (5) Å	µ = 0.26 mm^−^^1^
*c* = 11.2928 (9) Å	*T* = 173 K
β = 100.859 (7)°	Block, colourless
*V* = 2148.8 (3) Å^3^	0.39 × 0.26 × 0.25 mm
*Z* = 8	

### Data collection

**Table:**

Stoe IPDS-II two-circle diffractometer	2002 independent reflections
Radiation source: fine-focus sealed tube	1703 reflections with *I* > 2σ(*I*)
graphite	*R*_int_ = 0.040
ω scans	θ_max_ = 25.5°, θ_min_ = 3.4°
Absorption correction: multi-scan (*MULABS*; Spek, 2009; Blessing, 1995)	*h* = −29→29
*T*_min_ = 0.907, *T*_max_ = 0.939	*k* = −9→8
7423 measured reflections	*l* = −13→13

### Refinement

**Table:**

Refinement on *F*^2^	Primary atom site location: structure-invariant direct methods
Least-squares matrix: full	Secondary atom site location: difference Fourier map
*R*[*F*^2^ > 2σ(*F*^2^)] = 0.038	Hydrogen site location: inferred from neighbouring sites
*wR*(*F*^2^) = 0.103	H atoms treated by a mixture of independent and constrained refinement
*S* = 1.07	*w* = 1/[σ^2^(*F*_o_^2^) + (0.0572*P*)^2^ + 0.6628*P*] where *P* = (*F*_o_^2^ + 2*F*_c_^2^)/3
2002 reflections	(Δ/σ)_max_ < 0.001
163 parameters	Δρ_max_ = 0.20 e Å^−^^3^
35 restraints	Δρ_min_ = −0.30 e Å^−^^3^

### Special details

**Table:**

Geometry. All e.s.d.'s (except the e.s.d. in the dihedral angle between two l.s. planes) are estimated using the full covariance matrix. The cell e.s.d.'s are taken into account individually in the estimation of e.s.d.'s in distances, angles and torsion angles; correlations between e.s.d.'s in cell parameters are only used when they are defined by crystal symmetry. An approximate (isotropic) treatment of cell e.s.d.'s is used for estimating e.s.d.'s involving l.s. planes.
Refinement. Refinement of *F*^2^ against ALL reflections. The weighted *R*-factor *wR* and goodness of fit *S* are based on *F*^2^, conventional *R*-factors *R* are based on *F*, with *F* set to zero for negative *F*^2^. The threshold expression of *F*^2^ > σ(*F*^2^) is used only for calculating *R*-factors(gt) *etc*. and is not relevant to the choice of reflections for refinement. *R*-factors based on *F*^2^ are statistically about twice as large as those based on *F*, and *R*- factors based on ALL data will be even larger.

### Fractional atomic coordinates and isotropic or equivalent isotropic displacement parameters (Å^2^)

**Table:**

	*x*	*y*	*z*	*U*_iso_*/*U*_eq_	Occ. (<1)
S1	0.21599 (2)	0.61925 (7)	0.64064 (3)	0.0548 (2)	
N1	0.20667 (6)	0.55153 (18)	0.41220 (11)	0.0355 (3)	
H1A	0.1949 (7)	0.495 (2)	0.3461 (12)	0.041 (5)*	
H1B	0.2299 (7)	0.6339 (18)	0.4094 (17)	0.044 (5)*	
C1	0.19018 (6)	0.51441 (19)	0.51351 (12)	0.0307 (3)	
C2	0.14709 (6)	0.37457 (18)	0.51046 (12)	0.0280 (3)	
C3	0.09531 (7)	0.4503 (2)	0.55352 (17)	0.0346 (5)	0.817 (2)
H3A	0.1071	0.4995	0.6348	0.041*	0.817 (2)
H3B	0.0789	0.5406	0.4978	0.041*	0.817 (2)
C4	0.05109 (9)	0.3143 (4)	0.5574 (2)	0.0449 (6)	0.817 (2)
H4	0.0179	0.3640	0.5855	0.054*	0.817 (2)
C5	0.03278 (11)	0.2407 (4)	0.4326 (2)	0.0535 (7)	0.817 (2)
H5A	0.0040	0.1535	0.4346	0.064*	0.817 (2)
H5B	0.0159	0.3294	0.3760	0.064*	0.817 (2)
C6	0.08243 (15)	0.1650 (4)	0.3893 (3)	0.0553 (10)	0.817 (2)
H6	0.0697	0.1172	0.3068	0.066*	0.817 (2)
C7	0.12688 (9)	0.3016 (3)	0.38385 (17)	0.0424 (5)	0.817 (2)
H7A	0.1591	0.2529	0.3534	0.051*	0.817 (2)
H7B	0.1104	0.3916	0.3279	0.051*	0.817 (2)
C8	0.17150 (8)	0.2349 (3)	0.59767 (19)	0.0401 (5)	0.817 (2)
H8A	0.1842	0.2821	0.6793	0.048*	0.817 (2)
H8B	0.2044	0.1845	0.5709	0.048*	0.817 (2)
C9	0.07646 (14)	0.1784 (3)	0.6434 (2)	0.0466 (6)	0.817 (2)
H9A	0.0479	0.0913	0.6479	0.056*	0.817 (2)
H9B	0.0884	0.2263	0.7251	0.056*	0.817 (2)
C10	0.10857 (13)	0.0264 (3)	0.4743 (3)	0.0591 (7)	0.817 (2)
H10A	0.1415	−0.0210	0.4460	0.071*	0.817 (2)
H10B	0.0809	−0.0645	0.4756	0.071*	0.817 (2)
C11	0.12686 (11)	0.0991 (3)	0.6021 (2)	0.0493 (6)	0.817 (2)
H11	0.1429	0.0084	0.6594	0.059*	0.817 (2)
C3'	0.0918 (3)	0.4290 (11)	0.4335 (8)	0.037 (2)*	0.183 (2)
H3'1	0.0980	0.4610	0.3523	0.045*	0.183 (2)
H3'2	0.0774	0.5284	0.4703	0.045*	0.183 (2)
C4'	0.0475 (5)	0.2869 (14)	0.4214 (10)	0.046 (3)*	0.183 (2)
H4'	0.0109	0.3238	0.3721	0.055*	0.183 (2)
C5'	0.0714 (5)	0.1404 (17)	0.3637 (12)	0.040 (4)*	0.183 (2)
H5'1	0.0429	0.0503	0.3489	0.048*	0.183 (2)
H5'2	0.0794	0.1758	0.2846	0.048*	0.183 (2)
C6'	0.1241 (4)	0.0722 (14)	0.4391 (9)	0.043 (3)*	0.183 (2)
H6'	0.1372	−0.0283	0.3994	0.052*	0.183 (2)
C7'	0.1683 (3)	0.2123 (10)	0.4505 (8)	0.039 (2)*	0.183 (2)
H7'1	0.1757	0.2408	0.3696	0.046*	0.183 (2)
H7'2	0.2039	0.1727	0.5006	0.046*	0.183 (2)
C8'	0.1384 (3)	0.3213 (10)	0.6373 (7)	0.0338 (19)*	0.183 (2)
H8'1	0.1244	0.4179	0.6778	0.041*	0.183 (2)
H8'2	0.1749	0.2865	0.6862	0.041*	0.183 (2)
C9'	0.0414 (5)	0.2430 (18)	0.5496 (11)	0.057 (4)*	0.183 (2)
H9'1	0.0112	0.1585	0.5460	0.068*	0.183 (2)
H9'2	0.0295	0.3445	0.5885	0.068*	0.183 (2)
C10'	0.1166 (5)	0.0295 (15)	0.5663 (11)	0.045 (3)*	0.183 (2)
H10C	0.0897	−0.0645	0.5626	0.055*	0.183 (2)
H10D	0.1530	−0.0084	0.6136	0.055*	0.183 (2)
C11'	0.0957 (5)	0.1737 (16)	0.6295 (11)	0.041 (4)*	0.183 (2)
H11'	0.0892	0.1408	0.7112	0.049*	0.183 (2)

### Atomic displacement parameters (Å^2^)

**Table:**

	*U*^11^	*U*^22^	*U*^33^	*U*^12^	*U*^13^	*U*^23^
S1	0.0758 (4)	0.0683 (4)	0.0212 (2)	−0.0451 (3)	0.01175 (19)	−0.00661 (18)
N1	0.0424 (7)	0.0430 (8)	0.0228 (6)	−0.0139 (6)	0.0104 (5)	−0.0023 (6)
C1	0.0332 (7)	0.0362 (8)	0.0227 (7)	−0.0052 (6)	0.0053 (5)	0.0032 (6)
C2	0.0324 (7)	0.0308 (8)	0.0207 (6)	−0.0046 (6)	0.0048 (5)	0.0004 (5)
C3	0.0337 (9)	0.0358 (10)	0.0338 (10)	−0.0021 (8)	0.0052 (7)	−0.0008 (8)
C4	0.0373 (11)	0.0496 (16)	0.0490 (13)	−0.0057 (11)	0.0111 (9)	−0.0014 (11)
C5	0.0488 (15)	0.0577 (16)	0.0497 (15)	−0.0226 (13)	−0.0013 (11)	0.0048 (12)
C6	0.078 (2)	0.0540 (17)	0.0329 (14)	−0.0292 (15)	0.0086 (13)	−0.0115 (12)
C7	0.0573 (12)	0.0448 (12)	0.0262 (9)	−0.0168 (9)	0.0111 (8)	−0.0075 (8)
C8	0.0404 (11)	0.0374 (11)	0.0406 (11)	0.0012 (8)	0.0025 (8)	0.0096 (9)
C9	0.0510 (16)	0.0478 (15)	0.0425 (13)	−0.0167 (12)	0.0124 (12)	0.0020 (10)
C10	0.0797 (18)	0.0336 (13)	0.0666 (19)	−0.0123 (12)	0.0206 (15)	−0.0110 (12)
C11	0.0638 (15)	0.0329 (12)	0.0483 (13)	−0.0030 (11)	0.0032 (11)	0.0106 (11)

### Geometric parameters (Å, °)

**Table:**

S1—C1	1.6780 (14)	C9—H9B	0.9900
N1—C1	1.3149 (19)	C10—C11	1.542 (4)
N1—H1A	0.874 (9)	C10—H10A	0.9900
N1—H1B	0.870 (9)	C10—H10B	0.9900
C1—C2	1.5257 (19)	C11—H11	1.0000
C2—C3'	1.518 (7)	C3'—C4'	1.550 (12)
C2—C8	1.532 (2)	C3'—H3'1	0.9900
C2—C7	1.536 (2)	C3'—H3'2	0.9900
C2—C8'	1.546 (8)	C4'—C5'	1.507 (14)
C2—C3	1.552 (2)	C4'—C9'	1.524 (14)
C2—C7'	1.592 (8)	C4'—H4'	1.0000
C3—C4	1.533 (3)	C5'—C6'	1.498 (13)
C3—H3A	0.9900	C5'—H5'1	0.9900
C3—H3B	0.9900	C5'—H5'2	0.9900
C4—C9	1.509 (4)	C6'—C10'	1.521 (13)
C4—C5	1.515 (3)	C6'—C7'	1.538 (12)
C4—H4	1.0000	C6'—H6'	1.0000
C5—C6	1.509 (5)	C7'—H7'1	0.9900
C5—H5A	0.9900	C7'—H7'2	0.9900
C5—H5B	0.9900	C8'—C11'	1.561 (12)
C6—C10	1.523 (4)	C8'—H8'1	0.9900
C6—C7	1.543 (4)	C8'—H8'2	0.9900
C6—H6	1.0000	C9'—C11'	1.553 (14)
C7—H7A	0.9900	C9'—H9'1	0.9900
C7—H7B	0.9900	C9'—H9'2	0.9900
C8—C11	1.540 (3)	C10'—C11'	1.492 (13)
C8—H8A	0.9900	C10'—H10C	0.9900
C8—H8B	0.9900	C10'—H10D	0.9900
C9—C11	1.526 (4)	C11'—H11'	1.0000
C9—H9A	0.9900		
			
C1—N1—H1A	121.5 (13)	H9A—C9—H9B	108.1
C1—N1—H1B	120.4 (13)	C6—C10—C11	109.2 (2)
H1A—N1—H1B	118.0 (18)	C6—C10—H10A	109.8
N1—C1—C2	117.69 (13)	C11—C10—H10A	109.8
N1—C1—S1	120.42 (11)	C6—C10—H10B	109.8
C2—C1—S1	121.89 (11)	C11—C10—H10B	109.8
C3'—C2—C1	109.3 (3)	H10A—C10—H10B	108.3
C3'—C2—C8	140.3 (3)	C9—C11—C8	109.1 (2)
C1—C2—C8	109.80 (12)	C9—C11—C10	109.7 (2)
C3'—C2—C7	58.9 (4)	C8—C11—C10	108.4 (2)
C1—C2—C7	113.32 (13)	C9—C11—H11	109.9
C8—C2—C7	109.82 (15)	C8—C11—H11	109.9
C3'—C2—C8'	110.5 (4)	C10—C11—H11	109.9
C1—C2—C8'	113.1 (3)	C2—C3'—C4'	111.3 (7)
C8—C2—C8'	46.1 (3)	C2—C3'—H3'1	109.4
C7—C2—C8'	133.0 (3)	C4'—C3'—H3'1	109.4
C3'—C2—C3	52.3 (4)	C2—C3'—H3'2	109.4
C1—C2—C3	107.40 (13)	C4'—C3'—H3'2	109.4
C8—C2—C3	108.66 (14)	H3'1—C3'—H3'2	108.0
C7—C2—C3	107.70 (14)	C5'—C4'—C9'	110.3 (10)
C8'—C2—C3	63.8 (3)	C5'—C4'—C3'	106.9 (9)
C3'—C2—C7'	108.1 (5)	C9'—C4'—C3'	106.0 (9)
C1—C2—C7'	109.3 (3)	C5'—C4'—H4'	111.2
C8—C2—C7'	64.1 (3)	C9'—C4'—H4'	111.2
C7—C2—C7'	50.8 (3)	C3'—C4'—H4'	111.2
C8'—C2—C7'	106.3 (5)	C6'—C5'—C4'	113.1 (10)
C3—C2—C7'	142.7 (3)	C6'—C5'—H5'1	109.0
C4—C3—C2	110.17 (16)	C4'—C5'—H5'1	109.0
C4—C3—H3A	109.6	C6'—C5'—H5'2	109.0
C2—C3—H3A	109.6	C4'—C5'—H5'2	109.0
C4—C3—H3B	109.6	H5'1—C5'—H5'2	107.8
C2—C3—H3B	109.6	C5'—C6'—C10'	112.2 (10)
H3A—C3—H3B	108.1	C5'—C6'—C7'	106.9 (9)
C9—C4—C5	109.4 (2)	C10'—C6'—C7'	106.8 (8)
C9—C4—C3	109.02 (19)	C5'—C6'—H6'	110.3
C5—C4—C3	109.4 (2)	C10'—C6'—H6'	110.3
C9—C4—H4	109.7	C7'—C6'—H6'	110.3
C5—C4—H4	109.7	C6'—C7'—C2	110.6 (6)
C3—C4—H4	109.7	C6'—C7'—H7'1	109.5
C6—C5—C4	110.2 (2)	C2—C7'—H7'1	109.5
C6—C5—H5A	109.6	C6'—C7'—H7'2	109.5
C4—C5—H5A	109.6	C2—C7'—H7'2	109.5
C6—C5—H5B	109.6	H7'1—C7'—H7'2	108.1
C4—C5—H5B	109.6	C2—C8'—C11'	111.1 (6)
H5A—C5—H5B	108.1	C2—C8'—H8'1	109.4
C5—C6—C10	110.5 (3)	C11'—C8'—H8'1	109.4
C5—C6—C7	109.6 (2)	C2—C8'—H8'2	109.4
C10—C6—C7	109.2 (3)	C11'—C8'—H8'2	109.4
C5—C6—H6	109.2	H8'1—C8'—H8'2	108.0
C10—C6—H6	109.2	C4'—C9'—C11'	114.2 (10)
C7—C6—H6	109.2	C4'—C9'—H9'1	108.7
C2—C7—C6	109.51 (17)	C11'—C9'—H9'1	108.7
C2—C7—H7A	109.8	C4'—C9'—H9'2	108.7
C6—C7—H7A	109.8	C11'—C9'—H9'2	108.7
C2—C7—H7B	109.8	H9'1—C9'—H9'2	107.6
C6—C7—H7B	109.8	C11'—C10'—C6'	112.9 (9)
H7A—C7—H7B	108.2	C11'—C10'—H10C	109.0
C2—C8—C11	110.19 (15)	C6'—C10'—H10C	109.0
C2—C8—H8A	109.6	C11'—C10'—H10D	109.0
C11—C8—H8A	109.6	C6'—C10'—H10D	109.0
C2—C8—H8B	109.6	H10C—C10'—H10D	107.8
C11—C8—H8B	109.6	C10'—C11'—C9'	108.9 (10)
H8A—C8—H8B	108.1	C10'—C11'—C8'	109.2 (10)
C4—C9—C11	110.8 (2)	C9'—C11'—C8'	104.2 (9)
C4—C9—H9A	109.5	C10'—C11'—H11'	111.4
C11—C9—H9A	109.5	C9'—C11'—H11'	111.4
C4—C9—H9B	109.5	C8'—C11'—H11'	111.4
C11—C9—H9B	109.5		
			
N1—C1—C2—C3'	−66.3 (4)	C4—C9—C11—C8	60.1 (3)
S1—C1—C2—C3'	113.5 (4)	C4—C9—C11—C10	−58.4 (3)
N1—C1—C2—C8	120.45 (17)	C2—C8—C11—C9	−59.3 (2)
S1—C1—C2—C8	−59.78 (17)	C2—C8—C11—C10	60.1 (2)
N1—C1—C2—C7	−2.7 (2)	C6—C10—C11—C9	57.2 (3)
S1—C1—C2—C7	177.02 (13)	C6—C10—C11—C8	−61.8 (3)
N1—C1—C2—C8'	170.1 (4)	C1—C2—C3'—C4'	176.7 (6)
S1—C1—C2—C8'	−10.1 (4)	C8—C2—C3'—C4'	−13.3 (10)
N1—C1—C2—C3	−121.57 (16)	C7—C2—C3'—C4'	70.5 (7)
S1—C1—C2—C3	58.20 (16)	C8'—C2—C3'—C4'	−58.2 (8)
N1—C1—C2—C7'	51.9 (4)	C3—C2—C3'—C4'	−85.9 (7)
S1—C1—C2—C7'	−128.3 (3)	C7'—C2—C3'—C4'	57.8 (8)
C3'—C2—C3—C4	80.9 (4)	C2—C3'—C4'—C5'	−59.7 (10)
C1—C2—C3—C4	−177.77 (14)	C2—C3'—C4'—C9'	57.9 (10)
C8—C2—C3—C4	−59.06 (18)	C9'—C4'—C5'—C6'	−51.8 (15)
C7—C2—C3—C4	59.85 (19)	C3'—C4'—C5'—C6'	63.0 (13)
C8'—C2—C3—C4	−70.1 (4)	C4'—C5'—C6'—C10'	53.5 (15)
C7'—C2—C3—C4	12.4 (6)	C4'—C5'—C6'—C7'	−63.2 (13)
C2—C3—C4—C9	59.8 (2)	C5'—C6'—C7'—C2	58.7 (10)
C2—C3—C4—C5	−59.8 (2)	C10'—C6'—C7'—C2	−61.6 (9)
C9—C4—C5—C6	−59.6 (3)	C3'—C2—C7'—C6'	−57.6 (8)
C3—C4—C5—C6	59.8 (3)	C1—C2—C7'—C6'	−176.6 (6)
C4—C5—C6—C10	60.0 (3)	C8—C2—C7'—C6'	80.2 (6)
C4—C5—C6—C7	−60.3 (3)	C7—C2—C7'—C6'	−71.8 (6)
C3'—C2—C7—C6	−79.3 (4)	C8'—C2—C7'—C6'	61.0 (7)
C1—C2—C7—C6	−178.50 (19)	C3—C2—C7'—C6'	−6.9 (10)
C8—C2—C7—C6	58.3 (2)	C3'—C2—C8'—C11'	59.2 (8)
C8'—C2—C7—C6	10.5 (5)	C1—C2—C8'—C11'	−177.9 (6)
C3—C2—C7—C6	−59.9 (2)	C8—C2—C8'—C11'	−82.1 (7)
C7'—C2—C7—C6	85.0 (4)	C7—C2—C8'—C11'	−6.8 (9)
C5—C6—C7—C2	61.0 (3)	C3—C2—C8'—C11'	83.4 (7)
C10—C6—C7—C2	−60.2 (3)	C7'—C2—C8'—C11'	−57.9 (8)
C3'—C2—C8—C11	5.9 (6)	C5'—C4'—C9'—C11'	52.5 (15)
C1—C2—C8—C11	175.91 (18)	C3'—C4'—C9'—C11'	−62.8 (13)
C7—C2—C8—C11	−58.9 (2)	C5'—C6'—C10'—C11'	−55.3 (13)
C8'—C2—C8—C11	72.4 (4)	C7'—C6'—C10'—C11'	61.5 (11)
C3—C2—C8—C11	58.7 (2)	C6'—C10'—C11'—C9'	53.7 (13)
C7'—C2—C8—C11	−81.6 (4)	C6'—C10'—C11'—C8'	−59.5 (12)
C5—C4—C9—C11	59.2 (3)	C4'—C9'—C11'—C10'	−53.6 (14)
C3—C4—C9—C11	−60.5 (3)	C4'—C9'—C11'—C8'	62.9 (13)
C5—C6—C10—C11	−58.5 (3)	C2—C8'—C11'—C10'	58.0 (10)
C7—C6—C10—C11	62.1 (3)	C2—C8'—C11'—C9'	−58.2 (10)

### Hydrogen-bond geometry (Å, °)

**Table:**

*D*—H···*A*	*D*—H	H···*A*	*D*···*A*	*D*—H···*A*
N1—H1A···S1^i^	0.87 (1)	2.63 (1)	3.4027 (14)	148 (2)
N1—H1B···S1^ii^	0.87 (1)	2.49 (1)	3.3485 (14)	168 (2)

Symmetry codes: (i) *x*, −*y*+1, *z*−1/2; (ii) −*x*+1/2, −*y*+3/2, −*z*+1.
